# Highly conserved serine residue 40 in HIV-1 p6 regulates capsid processing and virus core assembly

**DOI:** 10.1186/1742-4690-8-11

**Published:** 2011-02-16

**Authors:** Jörg Votteler, Liane Neumann, Sabine Hahn, Friedrich Hahn, Pia Rauch, Kerstin Schmidt, Nicole Studtrucker, Sara MØ Solbak, Torgils Fossen, Peter Henklein, David E Ott, Gudrun Holland, Norbert Bannert, Ulrich Schubert

**Affiliations:** 1 Institute of Virology, Friedrich-Alexander-University, Erlangen, Germany; 2 Centre of Pharmacy, University of Bergen, Bergen Norway; 3 Institute of Biochemistry, Humboldt University, Berlin, Germany; 4 SAIC-Frederick, Inc., National Cancer Institute, Frederick, USA; 5 Robert Koch-Institute, Berlin, Germany

## Abstract

**Background:**

The HIV-1 p6 Gag protein regulates the final abscission step of nascent virions from the cell membrane by the action of two late assembly (L-) domains. Although p6 is located within one of the most polymorphic regions of the HIV-1 *gag *gene, the 52 amino acid peptide binds at least to two cellular budding factors (Tsg101 and ALIX), is a substrate for phosphorylation, ubiquitination, and sumoylation, and mediates the incorporation of the HIV-1 accessory protein Vpr into viral particles. As expected, known functional domains mostly overlap with several conserved residues in p6. In this study, we investigated the importance of the highly conserved serine residue at position 40, which until now has not been assigned to any known function of p6.

**Results:**

Consistently with previous data, we found that mutation of Ser-40 has no effect on ALIX mediated rescue of HIV-1 L-domain mutants. However, the only feasible S40F mutation that preserves the overlapping *pol *open reading frame (ORF) reduces virus replication in T-cell lines and in human lymphocyte tissue cultivated *ex vivo*. Most intriguingly, L-domain mediated virus release is not dependent on the integrity of Ser-40. However, the S40F mutation significantly reduces the specific infectivity of released virions. Further, it was observed that mutation of Ser-40 selectively interferes with the cleavage between capsid (CA) and the spacer peptide SP1 in Gag, without affecting cleavage of other Gag products. This deficiency in processing of CA, in consequence, led to an irregular morphology of the virus core and the formation of an electron dense extra core structure. Moreover, the defects induced by the S40F mutation in p6 can be rescued by the A1V mutation in SP1 that generally enhances processing of the CA-SP1 cleavage site.

**Conclusions:**

Overall, these data support a so far unrecognized function of p6 mediated by Ser-40 that occurs independently of the L-domain function, but selectively affects CA maturation and virus core formation, and consequently the infectivity of released virions.

## Background

The Gag polyprotein Pr55 of HIV-1 comprises the main structural components that are essential and sufficient for the formation of virus like particles (VLPs). Following synthesis in the cytoplasm, the Gag polyproteins are targeted to the plasma membrane where they assemble into immature budding particles. Concurrent with assembly and release of nascent virions, the Pr55 Gag precursor is cleaved by the autocatalytically activated viral protease (PR), generating the matrix (MA, p17), capsid (CA, p24), nucleocapsid (NC, p7), and the p6 protein. This processing ultimately leads to structural rearrangement of Gag molecules within the virion and the formation of the typical cone shaped core structure, characteristic for a mature infectious particle [1]. MA mediates the plasma membrane targeting of Gag polyproteins and, after cleavage, lines the inner shell of the mature virion. CA forms the conical core shell encasing NC, which regulates packaging and condensation of the viral genome [2-6]. The C-terminal p6 domain of Pr55, the smallest known lentiviral protein, containing 52 amino acids,, comprises a quite complex structural and functional organization and contains two distinct late assembly (L-) domains that regulate efficient separation of assembled virions from the cell surface. L-domains function as docking sites for components of ESCRT (endosomal sorting complex required for transport), cellular multi-protein complexes that are normally involved in the endocytotic recycling of cell surface receptors and in cytokinesis [4,7-17].

The L-domain activity of p6 is mainly driven by the ^7^PTAP^10 ^motif that is responsible for the recruitment of the primary budding factor Tsg101 (tumor susceptibility gene 101) to the virus assembly site [15,18-20]. Another region of p6 involves the residues ^36^YPLASL^41^, comprising a cryptic YPX_n_L-type L-domain, which forms a degenerated version of the YPDL L-domain motif (YPX_n_L, n = 3) found in equine infectious anemia virus (EIAV). This secondary L-domain in p6 represents a binding site for another cellular budding factor, AIP1/ALIX (ALG-2 interacting protein 1/X, ALIX is used hereafter), a multifunctional ESCRT-associated regulator of intracellular protein transport [13,21]. In addition to the interaction with cellular ESCRT components, p6 mediates the incorporation of the HIV-1 accessory protein Vpr into virus particles. This incorporation was shown to be dependent on three motifs in p6, the ^15^FRFG^18 ^motif, the ^34^ELY^36 ^motif, and the ^41^LXXLF^45 ^motif [22-24].

Besides these well characterized interactions, p6 contains several highly conserved amino acids, some of which were shown or proposed to undergo posttranslational modification. In this study, we show that mutation of the highly conserved Ser residue in position 40 (Ser-40) leads to an irregular core assembly of the released virions, a reduced infectivity, and, thus, a disturbed virus replication capacity. The results support a novel function of p6 in virus maturation that occurs independently of L-domain function.

## Results

### Mutation of Ser-40 has no effect on folding of p6

**Figure 1:**
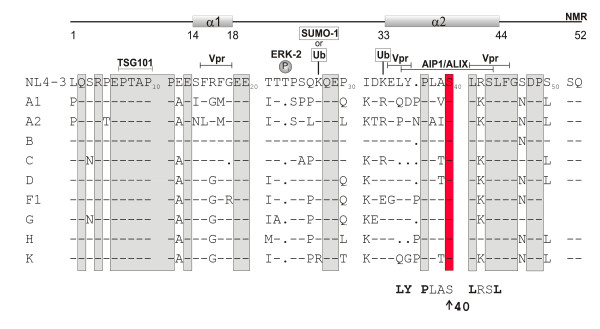
**Location and conservation of important functional sites in HIV-1 p6**. Primary sequence of p6 derived from the isolate HIV-1_NL4-3 _[28] and structural domains according to previous work [25]. Indicated are previously identified (ERK-2) phosphorylation sites [55], attachment sites for ubiquitin (Ub) [56] and SUMO-1 [57], and binding domains for Tsg101 [15,18,20,58], ALIX [13] and Vpr [22]. The consensus sequences of p6 proteins derived from the M-group viruses http://www.hiv.lanl.gov were aligned and conserved residues are boxed in grey.

HIV-1 p6 is located within one of the most polymorphic regions of the HIV-1 *gag *gene, yet the 52 amino acid peptide harbors one of the densest region of protein interacting domains in HIV-1. Figure 1A gives an overview of the previously reported binding domains of cellular (Tsg101, ALIX, ERK-2, SUMO-1, ubiquitin) and viral (Vpr) proteins within p6 from HIV-1_NL4-3 _and their relationship to the primary and secondary structures [25]. An alignment of consensus sequences, derived from all HIV-1 group M subtypes, revealed that the known functional domains overlap - as expected - with conserved residues in p6.

While the PTAP L-domain is highly conserved, the conservation within the ALIX binding site varies to some extent (Figure 1). The ALIX-binding motif has been defined as (L)[FY]PX_1-3_LXX[IL] [21,26,27] and corresponds in p6 derived from HIV-1_NL4-3 _[28] to ^35^**LYP**LAS**L**RS**L**^44^, in which essential residues are in bold. Interestingly, the three amino acid motif ^35^LYP^37 ^at the start of the binding region is only poorly conserved (Figure 1) while both, Leu-41 and Leu-44, are highly conserved. These two residues together, with the downstream Phe-45, comprise the LXXLF binding domain for the HIV-1 accessory protein Vpr. From previous structural and mutational analysis, it can be concluded that Thr-39 and Ser-40 do not directly participate in the binding of p6 to ALIX [21,27,29]. Consistently, Thr-39 is not conserved, while in contrast, Ser-40 is highly conserved among HIV-1 group M isolates, indicating a function other than ALIX binding.

To investigate a potential function of Ser-40 in p6, the residue was mutated in the infectious molecular clone HIV-1_NL4-3 _[28] and an otherwise isogenic R5-tropic derivative thereof carrying the 005pf135 V3 loop region in Env [30]. In order to obtain replication competent viruses, the mutation was introduced in a way that does not affect the overlapping *pol*-ORF. In particular, Ser-40 of p6 overlaps in the *pol*-ORF with the cleavage site between the transframe p6* protein and PR. The only possibility to leave the *pol*-ORF unaffected was to exchange Ser-40 for Phe, creating the mutant S40F.

**Figure 2:**
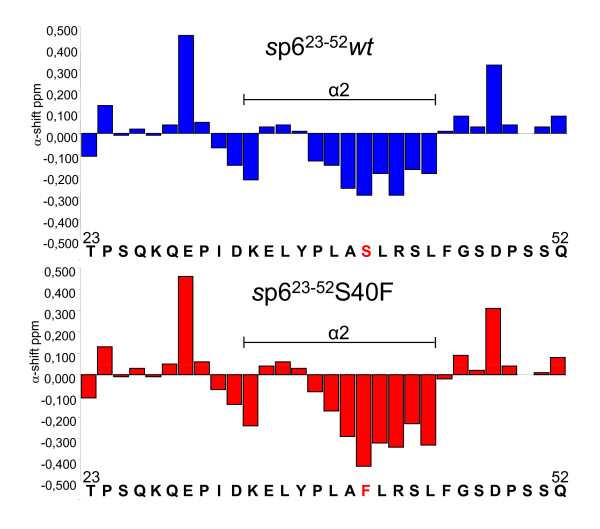
**Mutation of Ser-40 does neither affect the secondary structure of p6, nor the interaction with ALIX**. A) Chemical shift differences (ppm) of the α-protons between the experimental values and those for residues in a random coil for *s*p6^23-52^*wt *and *s*p6^23-52^S40F in 50% aqueous TFE at pH 3 at 300 K. All positive values for N-terminal residues adjacent to proline residues at positions 24, 30, 37 and 49 arise from an inherent effect of proline and not out of a structural perturbation. This was explained in detail previously [25].

Considering this limitation in mutating p6, we wanted proof that the non-conservative S40F exchange does not disturb the secondary structure of p6 in the respective region. To ascertain whether the S40F mutation alters the C-terminal helix of p6, the synthetic (*s*)p6^23-52^S40F peptide was characterised by ^1^H NMR spectroscopy [25]. Recently, we solved the structure of *s*p6^23-52 ^by NMR and found that the C-terminal fragment adopts the same structure as the full length molecule *s*p6^1-52^. Therefore, it was legitimate to analyse the structure of the mutant and compare it with the *wt *molecule. After complete assignment of the ^1^H resonances of the NMR spectra of the peptide, NOE cross peaks important for secondary structure identification were identified. The observation of NH_*i *_- NH_*i+1*_, NH_*i *_- NH_*i+2*_, αH_*i *_- NH_*i+ *__*2*_, αH_*i *_- NH_*i+ *__*3*_, αH_*i *_- NH_*i+ *__*4 *_and αH_*i *_- βH_*i + 3 *_NOEs, which are indicative of helical secondary structure, showed that, similarly to *wt s*p6^23-52^, *s*p6^23-52^S40F has a preference for an α-helical structure involving residues Ile-31 - Asp-48 under hydrophobic membranous conditions (50% aqueous trifluorethanol (TFE) solution). Substitution of Ser-40 with Phe did not change the position or number of residues included in the C-terminal helix compared with *wt s*p6^23-52^, and their structures appear to be similar. This is confirmed from a comparison of plots of the α-proton chemical shifts relative to those of random coil values (Figure 2), which indicates that the substitution slightly stabilizes the region of the C-terminal helix comprised of residues Leu-41 - Leu-44 proximal to Phe-40.

### Mutation of Ser-40 in HIV-1 compromises virus replication

**Figure 3:**
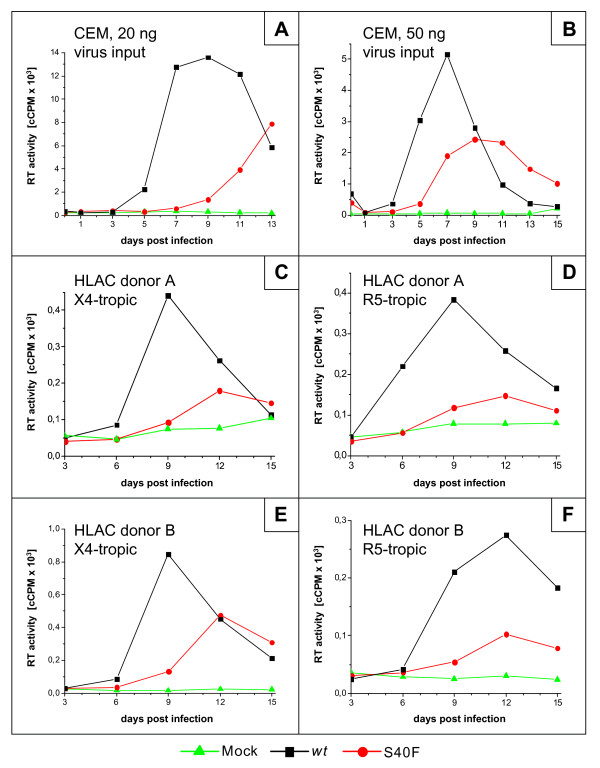
**Replication of HIV-1_NL4-3 _*wt *and S40F mutant in CEMs and HLAC**. CEM cells were inoculated with purified virus equivalent to 20 ng (A) or 50 ng (B) of p24 bearing indicated mutations, and medium was collected every two days. For replication analyses in HLAC, X4 tropic (C and E) and R5 tropic (D and F), 1 ng of p24 of purified virus carrying indicated mutations was used for infection and release of viral particles was determined in the supernatant every third day (C and D: donor A, E and F: donor B).

The finding that Ser-40 in p6 is highly conserved raises the question whether this amino acid is important for the function of p6 in HIV-1 replication. To investigate this, comprehensive replication studies of HIV-1 carrying mutation of Ser-40 in p6 were conducted in T cells and primary lymphocytes. Purified virus stocks of *wt *HIV-1_NL4-3 _and the S40F mutant were generated in 293T cells and normalized for p24 content. First, parallel cultures of CEM T-cells were infected with 20 and 50 ng of input virus, respectively, and samples of culture supernatants collected every other day were analyzed for secretion of virus particles by measuring the virus associated reverse transcriptase (RT) activity. The resulting replication profiles are shown in Figure 3A and 3B. Mutation of Ser-40 resulted in a diminished replication capacity of HIV-1, which is observed as a delayed onset of virus replication (Figure 3A). Furthermore, increase of the input virus to 50 ng resulted in a forward shift of the peak of virus replication. Yet, the overall virus production was still reduced, indicating altogether a compromised replication capacity of the Ser-40 mutant of p6 (Figure 3B). To further evaluate the relevance of Ser-40 in p6, replication studies were conducted in lymphoid cells, derived from human tonsillary tissue, cultivated as aggregate cultures (HLAC) [31]. The HLAC system has been reported to be of comparable value to that of previously described human lymphoid tissue cultures (HLT) where virus replication is studied in tissue blocks [32,33]. Generally, both HLACs and HLTs, support productive HIV-1 replication independently of the coreceptor tropism and do not require artificial exogenous activation of host cells [32,33]. Parallel cultures of HLAC were infected with X4-tropic or R5-tropic HIV-1_NL4-3 _carrying mutation of Ser-40. Samples of culture supernatant were harvested every third day, and secretion of viral particles was determined by measuring the virus associated RT activity. The resulting replication profiles obtained in tissue samples, derived from two different donors for X4-tropic strains, are shown in Figure 3C and 3E, and for R5-tropic variants in Figure 3D and 3F. Consistent with results obtained in CEM cells (Figure 3A, 3B), mutation of Ser-40 resulted in a reduced replication capacity of both X4-tropic and R5-tropic viruses.

### Mutation of Ser-40 in p6 does not affect virus release but disturbs CA maturation

**Figure 4:**
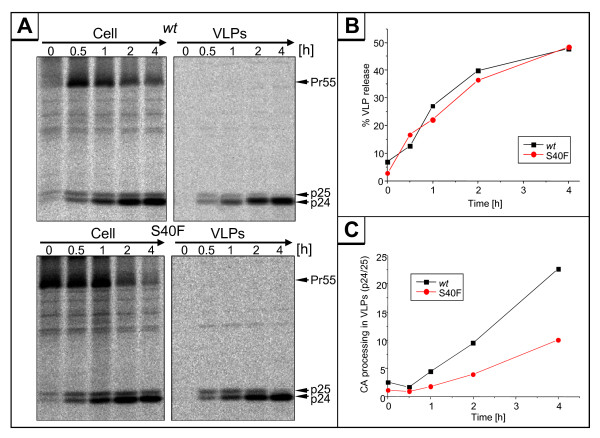
**Gag processing and release of virions**. (A) Phosphorimages of SDS-PAGE gels of immunoprecipitations of ^35^S pulse-chase-labeled Gag protein immunoprecipitates are presented for cell and viral lysates from HeLa cells transiently transfected with either pNLenv or pNLenv S40F. (B) Percentage of Gag released from the cell presented as the amount of Gag found in virus particles versus the total amount of Gag recovered form cell and virus lysates. (C) The rate of CA processing was estimated by calculating the ratio of mature CA p24 versus the CA precursor p25 detected in the cell and virus lysates at different time points.

To analyze whether the decreased replication capacity of the Ser-40 mutant is due to either a reduction of virus particle production or to a loss of infectivity of the released virions, we first investigated whether Ser-40 is somehow involved in the L-domain mediated assembly and release of virus particles. Virus release kinetics were studied by pulse chase metabolic labeling experiments. Parallel cultures of HeLa cells were transfected with the *env*-deleted HIV-1_NL4-3 _subgenomic expression vector pNLenv1 [34,35], encoding either *wt *p6 or the S40F mutant. Cells were pulse labeled with [^35^S]-methionine for 15 minutes and chased for up to 4 hours. At each time point indicated, samples of cells were harvested, and VLPs released into the supernatants were collected by centrifugation and processed for immunoprecipitation with Gag-specific antibodies. Immunoprecipitated proteins were separated by SDS-PAGE and analyzed by fluorography (Figure 4A). The amounts of Gag recovered from virus and cell lysates were quantified by image analysis. The release of VLPs was calculated as percentage of Gag proteins found in the virus fraction, relative to the total amount of Gag recovered from virus and cell fractions and plotted as a function of time (Figure 4B). Clearly, virus release was not affected by mutation of Ser-40. However, quantification of the CA processing products p25 and p24 revealed a significant reduction in the processing rate of p25 to p24 for the S40F mutant (Figure 4C). Thus, Ser-40 somehow regulates the proteolytic maturation of the CA-SP1 Gag processing intermediate, without affecting the overall efficiency of virus release.

### S40F mutation reduces specific infectivity of the virions

**Figure 5:**
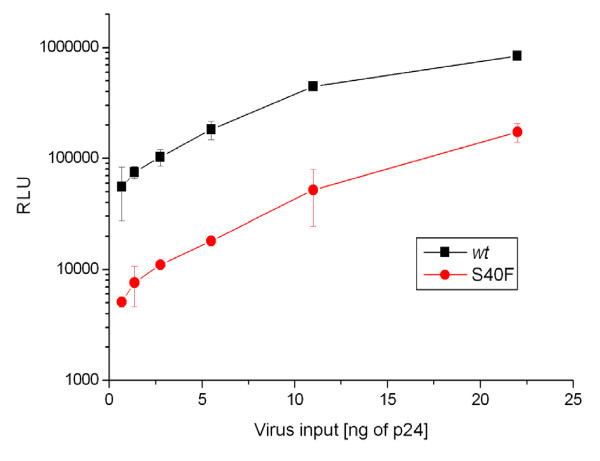
**Specific infectivity of HIV-1_NL4-3 _*wt *and p6-mutants**. TZM-bl cells were infected with purified virus preparations standardized for p24. Infectious titers were determined by measuring β-galactosidase activity as described in Materials and Methods (RLU: relative light units).

Having shown that mutation of Ser-40 in p6 does not affect L-domain mediated virus release but disturbs maturation of CA, we analyzed the role of this Ser residue in maturation of progeny virions. To measure the specific infectivity of the mutant, HeLa TZM-bl cells were infected with individual virus stocks standardized for p24 content and infectivity was determined by β-galactosidase assay. Consistent with virus replication data, mutation of Ser-40 reduced the infectivity by approximately 6-10 fold when compared to the *wt *virus (Figure 5).

### Mutation of Ser-40 has no effect on ALIX mediated virus release

**Figure 6:**
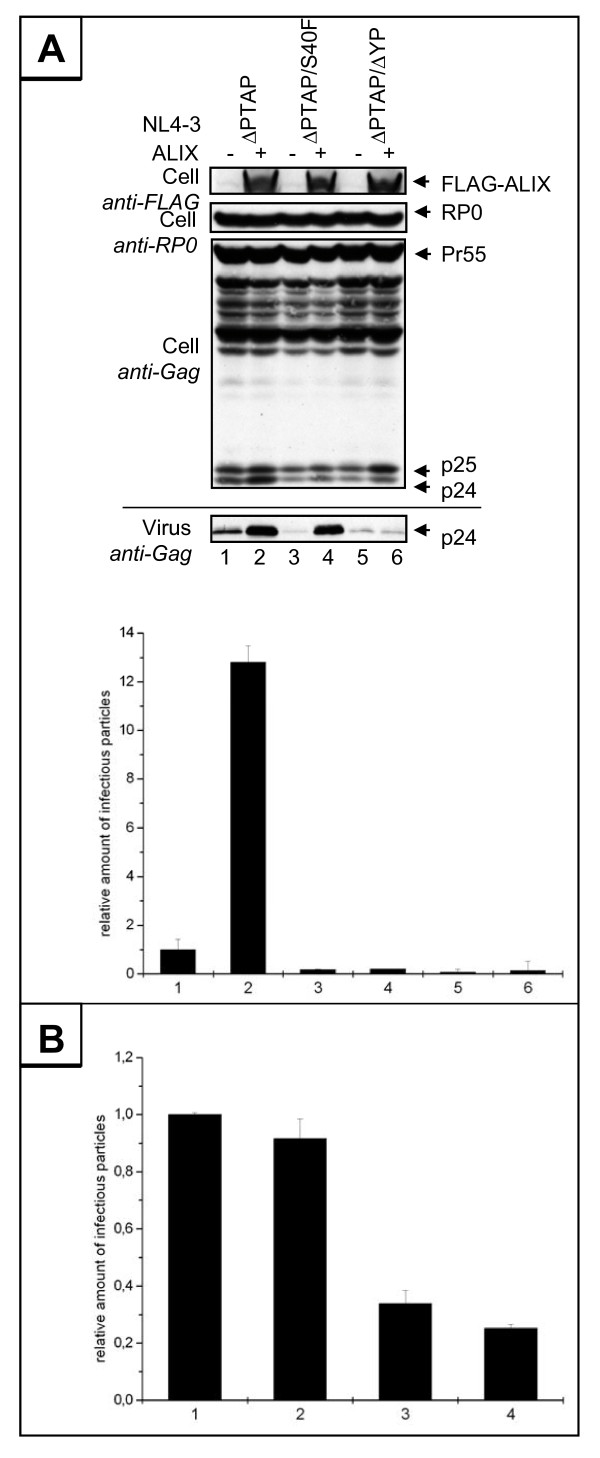
**S40F mutation has no effect on ALIX mediated virus release**. (A) Rescue of budding of the Ser-40 mutant by ALIX. Virus release, Gag processing, and exogenous expression of ALIX were analyzed by Western blot (upper panel). Ribosomal P0 antigen (RP0) was used as loading control. The amounts of infectious units released from the cells were analyzed by β-galactosidase quantification after infection of TZM-bl cells (lower panel). Shown are virus infectivities relative to the HIV-1_ΔPTAP _control ± SD. Lane 1 shows HIV-1_ΔPTAP _mutant cotransfected with the empty control vector, lane 2 shows HIV-1_ΔPTAP _mutant cotransfected with a FLAG-ALIX expression plasmid, lanes 3 and 4 show the HIV-1_ΔPTAP/ΔYP _double mutant cotransfected with the empty control plasmid and the vector expressing V5-POSH, respectively, lanes 5 and 6 show the HIV-1_ΔPTAP/ΔYP _double mutant cotransfected with the empty control plasmid and the vector expressing V5-POSH, respectively. (B) Overexpression of ALIX does not rescue infectivity of the S40F mutant. The amounts of infectious units released from the cells analyzed were by β-galactosidase quantification after infection of TZM-bl cells. Shown are virus infectivities relative to the HIV-1 control ± SD. 1: HIV-1 contransfected with the empty control vector, 2: HIV-1 cotransfected with a FLAG-ALIX expression plasmid, 3: HIV-1_S40F _cotransfected with the empty control vector, 2: HIV-1_S40F _cotransfected with a FLAG-ALIX expression plasmid.

Ser-40 residue is located within the previously identified ^36^YPLASL^41 ^ALIX binding sequence, although shown not to be involved in the interaction with ALIX directly [21,29]. It was further reported that mutation of either Leu-41 or Leu-44 directly adjacent to Ser-40 exhibit a similar phenotype to that observed for the S40F mutant in terms of CA processing [36]. Therefore, it was legitimate to investigate whether mutation of Ser-40 affects the functional interaction between ALIX and p6. We first examined the ability of ALIX to rescue the release of an HIV-1_ΔPTAP _L-domain mutant by overexpression in the absence of the Ser-40 [21,37]. The S40F mutation was cloned into a variant of HIV-1_NL4-3 _where the PTAP motif was replaced by LIRL (HIV-1_ΔPTAP _[38]). 293T cells were cotransfected with plasmids encoding ALIX and either HIV-1_ΔPTAP _or HIV-1_ΔPTAP/S40F_. As control, cells were cotransfected with HIV-1_ΔPTAP/ΔYP_, where the ALIX binding site YPX_3_L was replaced by SRX_3_L. As previously reported, this mutation completely abrogates ALIX mediated rescue of release of HIV-1_ΔPTAP _variants [21]. Gag processing and release of infectious virions were determined 24 hours post transfection by Western blot and single round infection of TZM-bl cells. The results shown in Figure 6A demonstrate that in the presence of the intact ALIX binding site in p6, overexpression of ALIX dramatically stimulated virus release by more than 10-fold as shown by Western blot and single round infection of TZM-bl cells (Figure 6A, lanes 1 and 2). Consistent with previous results [21,27], the mutation of the minimal ALIX binding motif further reduced virus release even below the residual budding of an HIV-1_ΔPTAP _mutant (Figure 6A, lane 5), and completely prevents rescue of the HIV-1_ΔPTAP _variant by overexpression of ALIX (Figure 6A, lane 6) [21]. Mutation of Ser-40 also reduced the residual release of the HIV-1_ΔPTAP _mutant (Figure 6A, lane 3). In contrast to the ΔYP mutation, in the presence of the S40F mutation, overexpression of ALIX still efficiently rescued the release of the HIV-1_ΔPTAP _mutant (Figure 6A, lane 4 upper panel). However, overexpression of ALIX did not restore the infectivity of the S40F mutant virions (Figure 6A, lane 4 lower panel), indicating that the loss of infectivity induced by mutation of Ser-40 occurs independently of the ALIX-p6 interaction.

To further uncouple the phenotype induced by S40F mutation from the underlying L-domain function of the ALIX binding site, we investigated whether ALIX can rescue the infectivity of the S40F mutant in the context of a functional PTAP motif. To this end, 293T cells were co-tranfected with HIV-1 encoding either *wt *p6 or the S40F mutant and ALIX. Release of infectious virions was determined 24 hours post transfection by single round infection of TZM-bl cells. Consistent with previous results, the S40F mutation reduced the infectivity of released virions by ~5-fold (Figure 6B, 1 and 3). While overexpression of ALIX had no significant influence on the infectivity of *wt *HIV-1 (Figure 6B, 2), ALIX also could not restore the reduced infectivity of the S40F mutant (Figure 6B, 4).

**Figure 7:**
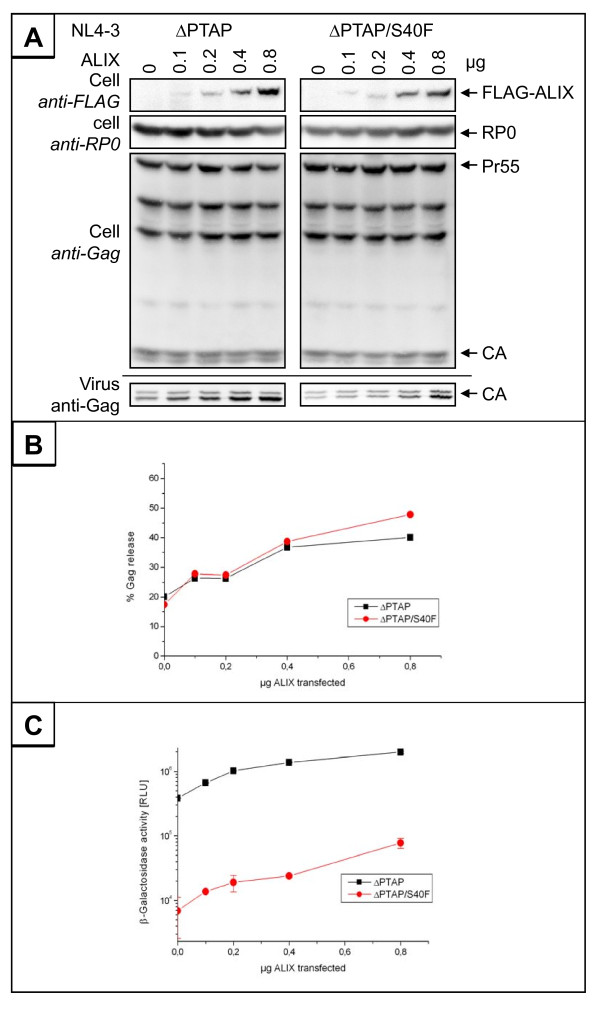
**Mutation of Ser-40 has no effect on ALIX mediated virus release**. (A) 293T cells were cotransfected with HIV-1_ΔPTAP _or HIV-1_ΔPTAP/S40F _and increasing amount of ALIX plasmid. (B) Quantification of Western blot data using AIDA Software (Raytest). Shown is the amount of Gag release determined by the ratio of virus associated Gag/total Gag expressed. (C) The amount of infectious units released from the cells was analyzed by β-galactosidase quantification after infection of TZM-bl cells.

In order to determine whether the ALIX mediated rescue of the HIV-1_ΔPTAP _variant of the S40F is still comparable to the control, we measured the requirement of ALIX for HIV-1_ΔPTAP _release at varying ALIX concentrations. 293T cells were cotransfected with HIV-1_ΔPTAP _and increasing amounts of ALIX expression plasmids. Subsequent determination of virus release by Western blot (Figure 7A) showed that in the presence of the S40F mutation similar amounts of ALIX were required to stimulate virus release (Figure 7A and 7B). Notably, ALIX substantially improved the processing of CA of the HIV-1_ΔPTAP _mutant. In contrast, CA processing of the HIV-1_ΔPTAP/S40F _mutant was further impaired, compared to the HIV-1_ΔPTAP _mutant, and was not rescued by overexpression of ALIX (Figure 7A). This was further supported by the notion that the infectivity of the S40F mutant virions was substantially reduced and could not be restored by overexpression of ALIX (Figure 7C). Taken together, the data indicate that the interaction of p6 with ALIX is not affected by replacing the conserved Ser-40 by Phe, and the phenotype induced by this mutation occurs independently of the ALIX mediated L-domain function of p6 in this region.

### The S40F mutation does neither affect cleavage of Gag products, other than CA, nor incorporation of Env

**Figure 8:**
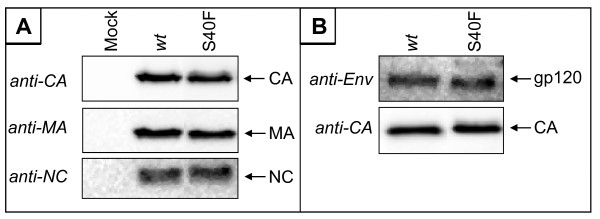
**Analysis of Gag processing products and Env incorporation in *wt *HIV-1 and S40F mutant virions**. (A) Analysis of Gag processing in VLPs. VLPs produced in 293T cells transiently transfected with pΔR and pΔR_S40F _were purified and analyzed by Western blot using antibodies against CA, MA and NC. (B) Analysis of Env incorporation in VLPs. VLPs produced in 293T cells transiently transfected with pΔR and pΔR S40F were purified and analyzed by Western blot using antibodies against Gag and Env.

In order to rule out the possibility that mutation of Ser-40 also affects processing of Gag proteins other than CA, virus preparations were analyzed by Western blotting using antibodies specific for NC or MA. Virus particles prepared by transient transfection of 293T cells were purified by centrifugation through a 20% sucrose cushion and standardized for p24 content by ELISA. Equal amounts of p24 were loaded on SDS-PAGE and analyzed by Western blotting. The amount of MA and NC proteins in S40F mutant viruses was similar to that of *wt *virions, indicating that maturation of these Gag proteins is not affected by the S40F mutation (Figure 8A). Even with the relatively low resolution of the SDS-PAGE system, the disturbed processing of CA from p25 to p24 was detectable (Figure 8A).

It was shown previously that mutations of p6 in this area, in particular, mutations of Tyr-36 and Leu-41 produce mutants that fail to package Env proteins into virus particles [39]. Since Ser-40 is located directly adjacent to Leu-41, we wanted to exclude that the reduced replication capacity and infectivity of the S40F mutant is due to reduced Env incorporation. To this end, purified virions standardized for p24 content were analyzed by Western blotting using Env specific antibodies. As shown in Figure 8B, the S40F mutation had no influence on Env incorporation into virus particles.

### Electron microscopy analysis of p6 S40F mutants

**Figure 9:**
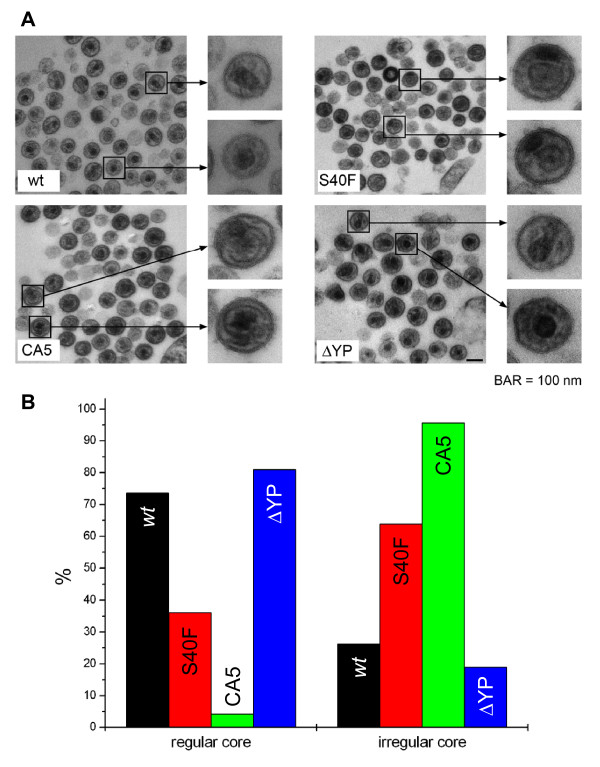
**Core morphology of wild type and mutant HIV-1 particles**. (A) Electron micrographs showing thin sections of HeLa SS6 derived extra cellular particles of HIV-1_NL-43 _*wt*, the S40F mutant, the CA-SP1 Gag cleavage deficient mutant CA5, and the ALIX binding site mutant ΔYP. A higher-magnification view of representative particles illustrates the dominating core structures. (B) Quantitative assessment of the relative amount of particles with regular and irregular core morphology. About 100-120 unselected particles of the *wt *virus and each mutant were evaluated. The data are representative for four independent experiments.

Next, we examined the effects of the S40F mutation on assembly, release, and virion morphology by thin-section electron microscopy (Figure 9). HeLa cells transiently transfected with plasmids encoding HIV-1_NL4-3 _and mutants thereof were drawn into cellulose capillary tubes 24 hours post transfection. The cellulose capillary tubes retain secreted virions, thereby obviating the need for centrifugation steps that usually affect the native virion structure. In agreement with our biochemical data, accumulation of virions tethered at the cell membrane, a phenotype commonly observed for L-domain mutants in p6, was not observed for the S40F mutant (data not shown). However, and most intriguingly, mutation of Ser-40 in p6 led to the formation of aberrant virus particles, characterized by irregularly shaped viral cores and the formation of closely neighboring electron-dense lateral bodies, as indicated in Figure 9A. These irregularly shaped cores and lateral bodies were not observed in the *wt *and the ΔYP mutant, again indicating that this phenotype occurs independently of the ALIX-Gag interaction. As we observed a deficiency in CA processing of p25 to p24 in the virions containing the S40F mutation (Figure 9), we investigated whether this defect in virus core assembly is a consequence of imperfect CA processing. A previously characterized CA5 mutant was generated in HIV-1_NL4-3 _that is incapable of processing p25 to p24 and was shown to exhibit a similar defect of core assembly [40]. Indeed, the phenotype observed for CA5 by analyzing the core structures of this mutant clearly resembles that of the S40F mutant (Figure 9A).

To evaluate quantitatively this morphological phenomenon, cores of 100 - 120 virons were counted and the percentages of irregular core structures relative to *wt *virions were calculated (Figure 9B). Obviously, mutation of Ser-40 significantly increases the amount of virions containing aberrant, irregularly shaped virus cores. Moreover, the CA5 mutant, in which CA processing is blocked completely, shows the same phenotype as that of the S40F mutant, further supporting the notion that Ser-40 governs the processing of CA by a yet unidentified mechanism.

### Defect in CA maturation of the S40F mutant can be rescued by mutation in the CA-SP1 cleavage site

**Figure 10:**
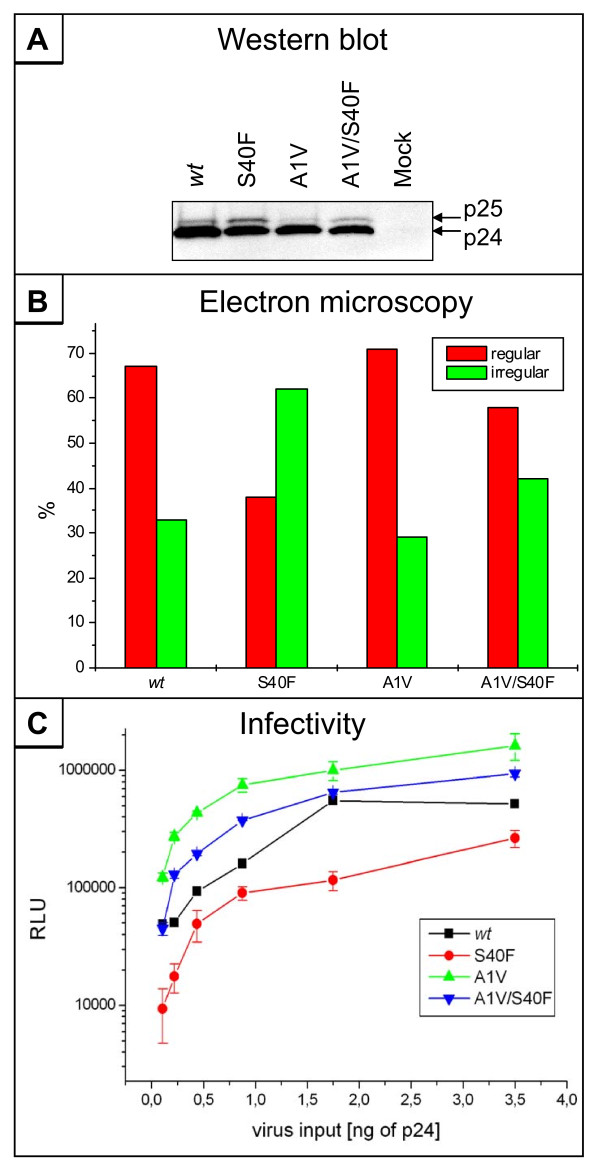
**Rescue of CA maturation by A1V mutation in CA-SP1 cleavage site**. (A) Western blot analysis of CA processing in VLPs. VLPs derived from 293T cells transfected with pΔR and pΔR_S40F _in combination with the A1V mutation in SP1 were purified and analyzed by Western blot using antibodies against CA. (B) Quantitative assessment of the viral core morphology derived from electron micrographs for the above indicated mutants. About 100-120 unselected particles of the *wt *virus and each mutant were evaluated. (C) Specific infectivity of HIV-1_NL4-3 _*wt *and S40F mutant in combination with the A1V mutation. TZM-bl cells were infected with virions derived from pNL4-3 *wt *and S40F in combination with SP1 A1V and infectious titers were determined by measurement of the β-galactosidase activity (RLU: relative light units).

As described above, the S40F mutation increases the ratio of p25 to mature p24. Our data, together with previous results from others, suggest that this disturbed CA processing subsequently leads to an irregular morphology of the virus core and thus, to reduced virus infectivity of the virions. This prompted us to investigate, whether restoring the CA processing by introducing specific mutations into the CA-SP1 cleavage site can rescue the defects in core assembly and infectivity induced by the S40F mutation. It is known that the affinity of the PR to the cleavage site between CA and SP1 is weak compared to other proteolytic cleavage sites in Gag [41,42]. The A1V mutation in the SP1, which previously was identified to confer resistance to the CA-maturation inhibitor Bevirimat [43], enhances the affinity of the viral protease to the CA-SP1 cleavage site. Thus, the A1V mutation in SP1 was introduced into the HIV-1_NL4-3 _backbone in combination with the S40F mutation in p6. Western blot analysis of purified virions revealed that, by introducing the A1V mutation, CA processing is substantially enhanced in both, the *wt *and the S40F mutant virions (Figure 10A). Furthermore, the A1V/S40F mutant displayed a similar CA processing compared to the *wt*.

Consequently, we wanted to examine whether this enhanced CA processing affects virus core assembly. Therefore, virion structure of the A1V mutants was analyzed by thin-section electron microscopy. Cores of 100 - 120 virons were counted and the percentages of irregular core structures relative to *wt *virions were calculated (Figure 10B). The S40F mutation again substantially increases the amount of virions containing aberrant, irregularly shaped virus cores. The A1V mutation had only marginal effects on the core morphology of *wt *HIV-1. However, in the case of the S40F mutant, introducing the A1V mutation largely improves virus core assembly (Figure 10B).

Since enhancing the CA processing rate rescues the defect of viral core assembly, we subsequently wanted to analyze whether this improved core formation also affects the infectivity of the virions. To measure the specific infectivity, HeLa TZM-bl cells were infected with individual virus stocks standardized for p24 content and infectivity was determined by β-galactosidase assay. The A1V mutation alone enhances the specific infectivity of the virons by 4-fold (Figure 10C). Introducing the A1V mutation enhances the specific infectivity of the virions of the otherwise attenuated S40F mutant to almost *wt *levels (Figure 10C), indicating that the deficiency in CA processing is the major determinant for the reduced infectivity of the S40F mutant.

## Discussion

In this study we demonstrate that mutation of the highly conserved Ser-40 interferes with Gag processing and virus core formation. Although Ser-40 in HIV-1 p6 is highly conserved among HIV-1 isolates, it is not involved in any of the functional motifs described so far, neither the ALIX nor the Vpr binding site. Therefore, it was legitimate to speculate that Ser-40 is involved in another, until now unrecognized function of p6. However, it should be noted that the position of Ser-40 in the nucleotide sequence of HIV-1 overlaps with the p6*/PR cleavage site in the overlapping *pol*-ORF, which limits the probability of mutations in this respective area and might contribute to the high conservation of this amino acid. Nevertheless, our findings of a compromised replication capacity and reduced infectivity of the Ser-40 mutant viruses support the assumption that Ser-40 has an important function directly associated with p6.

Notably, virus release kinetic was not reduced for the S40F mutant providing first evidence that the L-domain function of p6 is not affected by mutation of Ser-40. This was supported further by the observation that the ability of ALIX to rescue HIV-1_ΔPTAP _L-domain mutant viruses was not influenced by the S40F mutation. Although Ser-40 is located within the ALIX binding region in p6, previous structural investigations indicated that Ser-40 itself does not participate in the binding of p6 to ALIX [21,27,29]. The fact that the Ser-40 mutant was still fully active in terms of L-domain function further supports the notion, that the mutation introduced into p6 did not disturb the overall structure of the molecule in this respective region. In consistency, structural calculations indicated that the non-conservative exchange of Ser-40 to Phe does not change the ability of the molecule to adopt a helical structure. In fact, the C-terminal α-helix is conserved in the S40F mutant, as it was established by NMR studies of the C-terminal peptides *s*p6^23-52^.

Yet, as shown previously, the phenotypes induced by mutations in the ALIX binding site are depending on the type of amino acid that is mutated [36]. Mutation of the ^35^LYP^37 ^sequences in the ALIX binding site reduces release and infectivity of HIV-1 virions, which otherwise exhibit normal Gag processing [27,36]. In contrast, mutations of Leu-41 and Leu-44 have no impact on virus release but increase the ratio of p25 to mature p24, similar to the phenotype we observed for mutation of Ser-40 [36]. Unlike the typical phenotype of L-domain mutants, these mutants interfere somehow specifically with the final step in maturation of CA. It is currently not clear, whether this phenotype is in some way associated with the ALIX-p6 interaction. While mutations of Leu-41 and Leu-45 disrupt binding to ALIX, mutation of Ser-40 apparently has no influence on this interaction. Thus, it might also be possible, that this area in p6 harbors another function that, independent of L-domain activity, requires a so far unrecognized cellular interaction partner.

Maturation of the Gag processing intermediate p25 to mature CA p24, *e. g. *the cleavage of the CA-SP1 junction by the PR, appears to be one of the last steps of Gag processing [41,42]. It was previously demonstrated that mutating the junction between CA and SP1, in order to block cleavage of p25, leads to the production of noninfectious viral particles with aberrant core morphology [40]. In addition, treatment of virus producing cells with 3-O-(3'-3'dimethylsuccinyl) betulinic acid (BVM, Bevirimat, also known as PA-457 or DSB), a specific inhibitor that blocks PR-mediated cleavage between CA and SP1, disturbed viral core formation [43]. Intriguingly, mutation of Ser-40 leads to an almost identical aberrant virus core morphology as shown previously for CA5 mutants. Both are characterized by misshapen core structures and the formation of an electron dense lateral body near the viral membrane. Previous studies already indicated that maturation of HIV-1 virions, leading to the typical cone shaped cores, is regulated by the sequential, and highly ordered proteolytic cleavage of Gag [40]. Apparently, the last step of Gag processing - the cleavage of the CA-SP1 junction - is required for capsid condensation. However, pulse chase data indicate that the kinetic of cleavage of the CA-SP1 junction is delayed, but not completely blocked inasmuch as the mature CA accumulates over time. This indicates a rather dynamic process in which the mutation of Ser-40 somehow delays the kinetic of CA maturation. This phenomenon correlates with deficiencies in CA processing, infectivity, and core morphology.

Recently published results from Müller *et al. *demonstrate that even low amounts of Gag processing intermediates interfere with HIV particle maturation in a *trans*-dominant manner, with the final cleavage between p24 and SP1 being of particular importance [44]. This explains why the rather subtle effect on CA maturation detected for Ser-40 mutants by Western blotting and pulse chase analysis results in a substantial reduction of virus core formation.

Interestingly, the effect of the S40F mutation appears to be specific for the CA-SP1 cleavage inasmuch as i) no other Gag processing deficiency could be detected (Figure 6) and ii) enhancing CA processing by introducing the A1V mutation could restore the deficient core formation, and, consequently, enhance infectivity. Thus, it can be concluded that Ser-40 somehow regulates the cleavage of the CA-SP1 junction and the subsequent capsid condensation.

The molecular mechanism behind how Ser-40 regulates the processing of Gag, in particular the cleavage of the CA-SP1 junction, is still elusive so far. The previously described defects in Gag processing commonly observed for L-domain mutants are believed to be linked to the overall process of virus budding inasmuch as PR activation and subsequent Gag processing occur concomitantly with and shortly after release of virus particles [45,46]. In the case of Ser-40, this can be excluded, as the mutant S40F exhibits *wt *budding. However, Ser-40 in p6 and the CA-SP1 junction are separated by 123 amino acids in Pr55 and it remains elusive so far, how both proteins can affect each other, either in the context of Pr55 or after Gag processing. Our NMR experiments demonstrated that the C-terminal structure of p6 is not influenced by the S40F mutation. Therefore, one possibility of the effect observed would be that the mutation affects the Gag structure prior to initiation of Gag processing, thereby reducing the cleavage efficiency of the weakest cleavage site in Pr55. A prerequisite for this scenario would be that p6 represents a structured Gag domain and thus influence the folding the Pr55 polyprotein. Even though the 283 residue N-terminal part of HIV-1 Gag including MA and CA has been solved by NMR [47], the structure of the complete Pr55 has not been determined hitherto. Although S40F does not appear to affect the folding of the mature p6 protein, we can not exclude that this mutation indeed affect the overall structure of the PR55 polyprotein, which in turn would reduce the processing efficiency, a phenotype we clearly observed for the S40F mutant as a novel function of p6.

Currently, there is no evidence of an intra-molecular interaction between these domains in the Pr55 polyprotein. The p6 domain of the Pr55 represents a docking site of several cellular and viral factors. Thus, since an intramolecular interaction between CA and p6 appears to be unlikely, it is conceivable to hypothesize that p6 harbors another interaction domain of a yet unknown factor that, independently of the L-domains, regulates processing of CA.

## Conclusions

Overall, these data support a so far unrecognized function of p6 that occurs independently of the L-domain function, does not affect virus release, but selectively affects CA maturation, virus core formation, and thus, infectivity.

## Methods

### Peptide synthesis and purification

The synthesis, purification and molecular characterization of p6 and the related fragments derived from HIV-1_NL4-3 _have been described in detail previously [25].

### NMR Spectroscopy

2D^1^H Total Correlation Spectroscopy (TOCSY), Correlation Spectroscopy (COSY) and Nuclear Overhauser enhancement spectroscopy (NOESY) NMR experiments were performed at 600.13 MHz on a Bruker Avance 600 MHz instrument equipped with an UltraShield Plus magnet and a triple resonance cryoprobe with gradient unit. Individual samples were dissolved in 600 μl 50% aqueous TFE-d2 at concentrations between 1-2 mM. The 2D NMR experiments were performed at 300 K without spinning with mixing times of 110 ms for the TOCSY experiments and 250 ms for the NOESY experiments, respectively. Efficient suppression of the water signal was achieved with application of excitation sculpting in the 1D ^1^H and the 2D ^1^H TOCSY and NOESY NMR experiments. ^1^H signal assignments of the NMR spectra were achieved by identification of the individual spin systems in the 2D ^1^H TOCSY spectra, combined with observations of sequence-specific short-distance crosspeaks (H_α_-HN i, i+1) in the 2D ^1^H-^1^H NOESY spectra [48,49]. Readily recognizable spin systems were used as starting points for correlation of the individual spin systems observed in the TOCSY and NOESY spectra with individual residues in the peptide sequences. Acquisition of data, processing and spectral analysis were performed with Bruker Topspin 1.3 software.

### Antibodies

Antibody specific for FLAG was obtained from Sigma, the ribosomal P antigen specific antiserum from Immunovision Inc., the CA specific antiserum from Seramun. The p6 specific antibody was described earlier [25]. The anti-mouse, anti-rabbit, and anti-human IgG antibodies coupled to horseradish peroxidase (HRP) were obtained from Amersham.

### DNA mutagenesis

Amino acid exchanges at Ser-40 in p6 were introduced by site-directed mutagenesis using oligonucleotides containing the indicated changes (S40F, ΔYP, and ΔPTAP) and the Quick Change^® ^site directed mutagenesis kit (Stratagene). The mutations were introduced in the X4-tropic HIV-1_NL4-3 _infectious molecular clone [28] and isogenic R5-tropic derivative thereof [30]. In order to avoid taking biosafety measures, the mutations were also introduced in two HIV-1_NL4-3 _based subgenomic expression vectors giving rise to noninfectious VLPs: the pNLenv, in which *env *was deleted [50], and a an HIV-1 expression construct that carries a primer binding site deletion, as well as two point mutations in the active site of the RT coding region (pΔR [51]). All introduced mutations did not lead to mutations in the overlapping *pol*-ORF.

### Cell culture

HeLa SS6, HeLa TZM-bl and 293T cells were cultured in Dulbecco's modified Eagle's medium (DMEM) supplemented with 10% (v/v) inactivated fetal calf serum (FCS), 2 mM L-glutamine, 100 U/ml penicillin and 100 μg/ml streptomycin. CEM cells were maintained in RPMI 1640 supplemented with 10% (v/v) inactivated FCS, 2 mM L-glutamine, 100 U/ml penicillin and 100 μg/ml streptomycin. All media and compounds were provided by Gibco.

### Preparation and cultivation of primary cells

Human tonsils, removed during routine tonsillectomy, were received a few hours after excision from the Olgahospital, Stuttgart, Germany, prepared and infected as described earlier [32,33]. After washing the tonsils, human lymphocyte aggregate cultures (HLAC) were prepared by dividing the tonsils into tissue blocks of 2-3 mm and grinding the tissue through the sieve of a cell strainer (70 μm, BD Falcon) with a syringe plunger. Cells were seeded in a 96 well plate at a concentration of 2 × 10^6 ^cells per well. HLACs were cultured in RPMI 1640 supplemented with 15% (v/v) inactivated FCS, 2 mM L-glutamine, 100 U/ml penicillin and 100 μg/ml streptomycin, 2.5 μg/ml Fungizone, 1 mM sodium pyruvate, 1% (v/v) MEM non-essential amino acid solution and 50 μg/ml gentamicin.

### Western blot for protein analysis

HeLa SS6 cells were transiently transfected with the appropriate DNA using Lipofectamine 2000™ (Invitrogen) according to the manufacturer's protocol. For ALIX cotransfection, 293T cells were transfected with equal amounts of both DNAs and cells were harvested 24 h post transfection. Cells were lysed in cold RIPA buffer (50 mM Tris-HCl pH 7.4, 150 mM NaCl, 1% Nonidet P-40, 0.5% sodium deoxycholat, 0.1% Na-SDS, 5 mM EDTA, DNase, 1 mM PMSF and complete protease inhibitor cocktail (Boehringer Mannheim)), and the lysates were cleared by centrifugation at 16000 × *g *and 4°C for 10 min. RIPA-soluble proteins and VLPs were separated in 10% SDS/PAA gels, according to Laemmli [52], transferred onto PVDF membranes (GE Healthcare) and probed with specific antibodies, followed by enhanced chemiluminescence detection. For internal controls, blots were stripped and re-incubated with the appropriate antibody.

### Metabolic labeling and immunoprecipitation

For pulse chase experiments, adherent cultures of transfected HeLa SS6 cells were washed once with PBS and starved for 30 min in methionine-free, serum-free RPMI 1640. Cells were pulse-labeled for 15 min with [^35^S]-methionine (3 mCi/ml; Amersham Life Sciences) and chased for up to 4 h while shaking at 37°C in D-MEM, supplemented with 10% FCS and 10 mM methionine. At the indicated time points, cells and supernatants were collected by centrifugation for 1 min at 16000 × *g*. Virions were pelleted through a 20% (w/v) sucrose cushion and lysed in Triton wash buffer (50 mM Tris-HCl pH 7.4, 300 mM NaCl, 0.1% Triton X-100, 1 mM PMSF). Cells were lysed in RIPA buffer as described above, containing additionally 5 mM N-ethylmaleimide and 20 μM carbobenzoxyl-Leu-Leu-leucinal (zLLL; Sigma). Gag proteins from precleared cell lysates and lysed VLPs were recovered by immunoprecipitation using a mixture of polyclonal rabbit anti-p6 and anti-p24 antibodies prebound to protein G-Sepharose (GE Healthcare). Samples were separated by SDS-PAGE on a 10% (w/v) acryl amide ProSieve gel (Cambrex Bioscience), backed with Gel Bond film (FMC Bioproducts). Following fixation for 1 h in 50% methanol and 10% acetic acid, gels were rinsed with water, soaked in 1 M sodium salicylic acid solution with 10% glycerol for 5 h and dried. Radioactivity in dried gels was quantified using AIDA imaging software (Raytest).

### Viruses

Virus containing cell culture supernatant was harvested after 48 h and, after removal of residual cells by centrifugation, passed through a 0.45 μm pore-size filter. Virus was pelleted through 20% (w/v) sucrose (16000 × *g*, 4°C, 90 min). Virus stocks were normalized for p24 content as quantified by a enzyme-linked immunosorbent assay (ELISA, Aalto, Dublin, Ireland) and aliquots were stored at -80°C.

### Infection of cells

For infection of T cell cultures, 1 × 10^7 ^cells were incubated with 20 or 50 ng of p24, respectively, and supernatant was collected every second day post infection. Virus replication was assessed by quantification of the virus-associated RT activity by [^32^P]-TTP incorporation using an oligo(dT)-poly(A) template as described [53]. For testing each virus in the HLAC from one donor, 1 ng of p24 was applied to 2 × 10^6 ^cells in 96 well format, and virus replication was assessed, as described for T cell cultures, every third day post infection.

### Viral infectivity assay

HeLa TZM-bl cells were seeded in 96 well format (4000 cells per well) and infected with standardized amount of p24. The next day, fresh medium with 100 μg/ml dextran sulphate was added to prevent further spread of virus infection, and cells were incubated for further two days. Infection was detected using a galactosidase screen kit from Tropix as recommended by the manufacturer. β-Galactosidase activity was quantified as relative light units per second using an Orion Microplate Luminometer (Berthold).

### Transmission electron microscopy (TEM)

Transfected HeLa SS6 cells were processed for transmission electron microscopy in the following way: 24 h post transfection, cells were placed in cellulose capillary tubes [54], cultivated for one more day, then fixed in 2.5% glutaraldehyde for 1 h at 37°C and stored for further preparation at 4°C. Tubes were collected by centrifugation and sealed by immersion in low-melting-point agarose. The samples were post fixed with OsO_4 _(1% in distilled water, 1 h), tannic acid (0.1% in Hepes 0,05 M, 30 min) and uranyl acetate (1% in distilled water, 2 h) followed by stepwise dehydration in a graded ethanol series and embedding in epon resin, which was subsequently polymerized. Thin sections were prepared with an ultramicrotome (Ultracut S; Leica, Wetzlar, Germany) and counterstained with uranyl acetate and lead citrate. The sections were examined using a TEM 902 (Carl Zeiss SMT AG) at 80 kV, and the images were digitized using a slow-scan charge-coupled-device camera (Pro Scan; Scheuring, Germany). The evaluation of the capsid morphology was performed by using these images or directly on the screen.

## Competing interests

The authors declare that they have no competing interests.

## Authors' contributions

US designed the study. LN, SH, FH, PR, KS, NS and JV performed virus replications, infectivity as well as Western blot and pulse chase analysis of virions. SMØS and TF performed NMR studies, PH synthesized the peptides and DEO supplied essential material. NB and GH did the electron microscopy. JV and US wrote the manuscript. All authors read and approved the final manuscript.
